# *PpLEA* genes in embryophytes,instead of *LEAs* being angiosperm-unique

**DOI:** 10.3389/fpls.2026.1917865

**Published:** 2026-09-15

**Authors:** Jing Du, Ji Li, Lei Wang

**Affiliations:** Key Laboratory of Xinjiang Phytomedicine Resource and Utilization of Ministry of Education, Xinjiang Production and Construction Corps Key Laboratory of Oasis Town and Mountain-basin System Ecology, College of Life Sciences, Shihezi University, Shihezi, China

**Keywords:** Angiospermae, embryophytes, gametophore, late abundant embryogenesis protein, *Physcomitrella patens*, PpLEA, sporophyte

## Abstract

From the earliest land plants (Bryophyta) to the highly diversified flowering plants (Angiosperm), late embryogenesis abundant (*LEA*) genes/proteins are consistently identified across embryophytes. LEA proteins are intrinsically disordered proteins that are spatiotemporally expressed in floral organs/fruits or specifically somatic embryogenesis among various cell types. Moreover, *LEA* genes are typically responsive to various environmental stressors. To disclose the multifaceted features from vegetative to productive processes, this mini-review summarizes current knowledge on PpLEA genes/proteins in *Physcomitrella patens* (*P. patens*)-- a representative ancestral bryophyte land plant. In *P. patens*, LEA proteins maintain basal expression levels in protonema, probably due to the sufficient water supply under normal growth conditions; comparably those proteins are significantly accumulated in mature gametophores, consistent with a dehydration-protective function, revealing their extensive roles throughout development progression. While a majority of *PpLEA* genes consistently expressed across gametophyte-to-sporophyte transition, indicating their putative role linking vegetative and reproductive development. Only one single *PpLEA* gene showing differential transcriptional expression during the gametophyte to sporophyte transition is activated at this developmental onset and maintains the expression thereafter, suggesting its unique and non-redundant function linking vegetative and reproductive developmental stages. In addition, *P. patens* biosynthesize distinct LEA proteins in response to drought, chilling or abscisic acid (ABA) and the other abiotic stimulus. Therefore, the roles of *PpLEAs* can be further exploited in multifaceted ways. These findings shed light on the linkage between abiotic stress responses and plant development processes, particularly the transition from vegetative growth to reproductive development.

## 1 Introduction

Approximately 470–450 million years ago, the ancestral plants were gradually landing from water (Barthlott et al., 2016; Moody, 2020). During this terrestrialization event, early land plants underwent dramatic environment changes and evolved extensive physiological adaptations to cope with water-limited terrestrial habitats, gradually establishing vascular plants as the primary terrestrial vegetation to dominate global ecosystems (Silvestro et al., 2015). Under conditions mimicking the terrestrial landing environment, including abiotic stresses such as drought, freezing, salinity and heat, LEA proteins significantly accumulate in the vegetative tissues of resurrection plants (Hincha and Thalhammer, 2012; Leprince et al., 2017). As an ancestral non-vascular land plant of the division Bryophyta, *P. patens* produce filamentous protonema during vegetative phase. Subsequently it develops reproductive leafy-like gametophore through asymmetric cell division, which is generated from filamentous protonema (Kosetsu et al., 2017). Transcriptomic and proteomic profiling approaches have been utilized to characterize *PpLEA* genes and proteins in protonema and gametophore, revealing their diverse expression patterns across developmental stages (Fesenko et al., 2015, Fesenko et al., 2017), and dozens- to hundreds-fold higher abundance (**Supplementary Table 1**) in gametophores (Fesenko et al., 2015). Short-day photoperiod and low-temperature stimuli can induce the transition from the gametophyte to early and mid-sporophytes. Meanwhile, most of *PpLEA* genes represented no detectable expression differences during this development-to-reproduction transition, which indicates that this drastic environmental change exerts unexpectedly limited impacts on PpLEAs (O'Donoghue et al., 2013). The phenomenon indicates that *PpLEAs* exert diverse functions, acting not only in protection against stresses, but also playing roles during reproductive development. Accumulating evidence shed light on the dual role of *PpLEAs* in linking embryogenesis with abiotic stress responses (Smolikova et al., 2020; Wu et al., 2025). Although the functions of PpLEA proteins remain not fully elucidated, a consensus has emerged that members of this protein family play a vital role in stress tolerance and productive tissue maintenance.

## 2 The distribution of LEA genes/proteins among embryophytes

Late embryogenesis abundant (LEA) proteins were first identified during seed maturation in cotton (Dure and Galau, 1981), with rapid accumulation during late embryonic development. From the earliest land plants (Bryophyta) (Jahan et al., 2019; Penaloza-Bojaca et al., 2025) to the diversification of flowering plants (Angiospermae) (Hundertmark and Hincha, 2008; Gao and Lan, 2016; Liu et al., 2019), LEA genes and proteins are consistently and widely identified in a majority of embryophytes (Artur et al., 2019). However, early land plant ancestors faced severe selective pressures in terrestrial habitats (Kenrick and Crane, 1997), including intermittent water limitation during vegetative and reproductive growth under fluctuating seasonal conditions (Wang et al., 2020). Consequently, the life cycle of land plants has evolutionarily diverged into two distinct stages: the haploid gametophyte for vegetative growth and diploid sporophyte for reproductive development (Lueth and Reski, 2023).

To reveal the role of PpLEA proteins in moss against diverse stress conditions, multiple molecular-genetic approaches have been utilized (**Figure 1**): for instance, knockout of negative regulators results in the induction of most PpLEA proteins *in vivo* (Komatsu et al., 2013); furthermore, rice (Li et al., 2015) and *Arabidopsis thaliana* (Du et al., 2016; Li et al., 2017) have been used as heterologous planta systems to assess the PpLEA function against salinity and drought stresses; conversely, ectopic expression of an *Arabidopsis* negative regulator of ABA-signaling in *P. patens* modulates downstream *PpLEA* responses (Komatsu et al., 2009). Besides well-documented roles of LEA proteins in enhancing plant tolerance to various abiotic stresses (Rosete-Enriquez et al., 2025), emerging studies have highlighted their functions in plant developmental regulation. For instance, Group 4 LEA proteins in *Arabidopsis* are persistently expressed during embryogenesis, germination and seedling establishment. Furthermore, the stable and gradual accumulation of PpLEA4 in reproductive organs indicates these proteins primarily function to protect progeny tissues, instead of acting only in vegetative-tissue development (Olvera-Carrillo et al., 2010).

**Figure 1:**
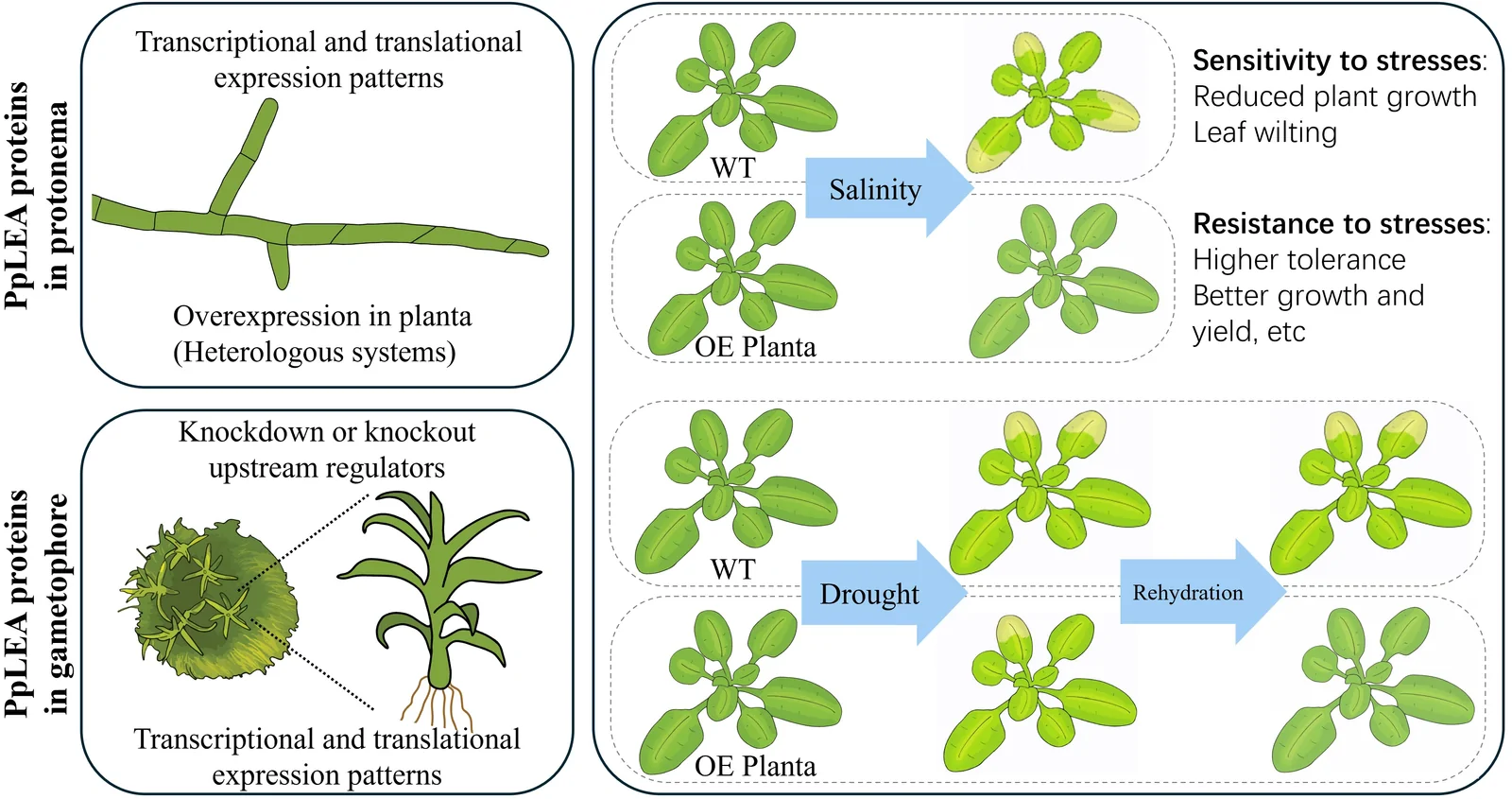
The resistance role of LEA proteins to stress conditions in heterologous expression transgenic lines.

Transcriptional (Oliver et al., 2004; Minami et al., 2005; Cuming et al., 2007; Shinde et al., 2012, Shinde et al., 2013) and translational (Wang et al., 2009; Cui et al., 2012; Fesenko et al., 2015) profiling approaches have been extensively applied to explore the role of *PpLEA* genes and proteins. Collectively, these findings emphasize the adaptive functions of LEA proteins against environmental stresses, while recent investigations further provide novel insights into their evolutionary significance (Artur et al., 2019; Szlachtowska and Rurek, 2023). Exploring the functional characteristics of LEA proteins across different plant species advances our understanding of their biological roles. Beyond facilitating plant tolerance against diverse stresses at the cellular and organ levels, LEA proteins may also contribute to elucidating the evolutionary origin of multicellular productive organs.

## 3 *In vivo* evidences of LEA genes/proteins during seed development/embryogenesis

Comparative multi-omics analyses reveal the multifaceted roles of LEA genes/proteins in embryogenesis or seed development (Fesenko et al., 2015; Leprince et al., 2017; Smolikova et al., 2020). Those LEA proteins are intrinsically disordered proteins spatiotemporally expressed in floral organs/fruits (Lin et al., 2024; Hou et al., 2025) or specifically induced during somatic embryogenesis (Li et al., 2023) among the various cell types.

The existence of LEA proteins/genes across all major land plant species, from bryophyta to agiospermae, has been clarified and summarized in **Table 1**. Based on phylogenetic and synteny analyses of *LEA* genes from 60 diverse whole-genome species, ranging from green algae to angiosperms, LEA homologs could be classified into eight multigene subfamilies: Dehydrin, LEA_1, LEA_2, LEA_3, LEA_4, LEA_5, LEA_6 and seed maturation protein (SMP). Among all subfamilies, LEA_5 and SMP appear to be present in early-diverging plant lineage, whereas the other LEA subfamilies show at comparably later evolutionary stages (Artur et al., 2019). To emphasize the key evolutionary shift associated with plant transition from freshwater to terrestrial habitats, where plants are persistently exposed to severe water deficit, the LEA subfamilies gradually expanded and diversified within embryophytes. While the significant variation in their gene copy numbers (**Table 1**) reflects the dynamic and complex evolutionary track of LEA family.

**Table 1:** Plants LEAs in embryophytes.

Embryophytes		LEA/LEA-like proteins (Yes or No)	Description		
Classification Typical species			Characteristics	Stress responses	References
B R Y O P H Y T A	Hepaticae Liverwort (*Marchantia polymorpha*)	Yes 35 LEA-like proteins	Transcriptional expression induced by ABA	ABA induces accumulation of LEA-like transcripts and soluble sugars	(Jahan et al., 2019)
	Musci Moss *(Physcomitrella patens)*	Yes LEA proteins and LEA-like proteins	Dehydration might cause transcriptional oscillatory increase in various developmental stages	The expression of PpLEA proteins enhanced during drying	(Minami et al., 2005; Cuming et al., 2007; Cui et al., 2012; Yotsui et al., 2016; Artur et al., 2019; Yuen et al., 2019)
	Anthocerotophyta Hornwort (*Anthoceros angustus*)	Undetermined			
PTERIDOPHYTA Filicinae, Lycopodiinae, Equisetinae		Undetermined			
G Y M N O S P E R M A E	Pinopsida *Pinus tabuliformis*	Yes 23 LEA genes in seven groups	Protection against stress conditions at cellular level	an increased resistance to heat and salt, but not significantly	(Gao and Lan, 2016)
	Cycadopsida	Undetermined			
	Ginkgoopsida				
A N G I O S P E R M A E	Monocotyledonous *Triticum aestivum*	Yes 179 LEA genes in eight groups	ABA and abiotic stress responsive cis-acting elements in the promoter region	Protection against heat and salt stress	(Liu et al., 2019)
	Monocotyledoneae *Zea mays*	Yes 52 LEA genes in seven groups	Participating in the regulation of the kernel (KDR) dehydration rate	Responsive to dehydration	(Yuen et al., 2019; Zhang et al., 2023)
	Monocotyledoneae S*orghum bicolor L.*	Yes 68 LEA genes in eight groups	Biotic stress-responsive and development-responsive cis-elements enriched in the promoter of LEA genes	Up-regulation under different stress conditions	(Nagaraju et al., 2019)
	Monocotyledoneae *endrobium officinale*	Yes 17 LEA genes in seven groups	Quickly responsive to abiotic stresses	Up-regulation under salt and heat stresses	(Ling et al., 2016)
	Monocotyledoneae C*leistogenes songorica*	Yes 44 LEA genes in eight groups	Transcriptional response to heat stress and ABA treatment	CsLEA2 genes enhancing plant abiotic stress tolerance	(Muvunyi et al., 2018)
	Monocotyledoneae *Setaria italica L.*	Yes 94 LEA genes in nine groups	Involved in plant growth regulation, stress response, and hormone signaling	Transcriptional induction by ABA	(Qin et al., 2025)
	Monocotyledoneae *Phyllostachys edulis*	Yes 23 LEA genes in six groups	*PeLEAs* may have occurred around 18.8 million years ago	*PeLEAs* induced by dehydration and cold stresses	(Huang et al., 2016)
	Monocotyledoneae *Ananas comosus L.*	Yes 37 LEA genes in six groups	*AcLEA* genes spatiotemporally expressed in floral organs/fruits, most of the gene induced by cold stress	Transcriptional upregulated in cold	(Hou et al., 2025)
	Dicotyledoneae *Arabidopsis thaliana*	Yes 51 LEA genes in nine groups	The genes predicted to be highly hydrophilic and natively unstructured	Most LEA genes had abscisic acid response and/or low temperature response elements in their promoters	(Hundertmark and Hincha, 2008)
	Dicotyledoneae *Liquidambar*	Yes 79 LEA genes in eight groups	Significant specificity expression during somatic embryogenesis	Abiotic stresses and light-responsive elements enriched in *LsfLEA* gene promoters	(Hundertmark and Hincha, 2008)
	Dicotyledoneae *Vitis amurensis*	Yes 52 LEA genes in seven groups	Enhancement of grapevine’s tolerance against cold and osmotic stresses (*VamDHN3* gene)	Responsive to cold and osmotic stresses	(Xu et al., 2020)
	Dicotyledoneae *Arachis hypogaea* L.	Yes 126 LEA genes in eight groups	Conserved exon-intron and motif structure of grouping *AhLEAs* responsive to abiotic stressors	Enhancement of AhLEA1, AhLEA3–1 and AhLEA3–3 proteins to cold and aluminum tolerance; protective role of AhLEA3–1 against drought	(Huang et al., 2022)
	Dicotyledoneae *Ramonda serbica* Panc	Yes 318 LEA genes in seven groups	Structural characterization of LEA proteins: hydrophilic LEA4 proteins to form amphipathic α-helices; LEA2 containing globular domains. highly disordered DEH1, LEA1, LEA4.2, and LEA4.3 proteins	Undetermined	(Pantelic et al., 2022)
	Dicotyledoneae *Capsicum annuum L.*	Yes 82 LEA genes in nine groups	Major LEA genes expressed during embryonic development and stress response	Particularly responsive to chilling stress	(Zhao et al., 2023)
	Dicotyledoneae *Camellia sinensis*	Yes 48 LEA genes in seven groups	Dominant expression in the root or seed, especially during tea seed maturation concurrent with dehydration	Especially sensitive to high temperature stress	(Liu et al., 2019)
	Dicotyledoneae *Notopterygium* species: *Notopterygium. franchetii; Notopterygium. Incisum; Notopterygium forrestii*	Yes 20–23 LEA genes in six groups	20–23 LEA genes among three *Notopterygium* species, exhibited significant variations in the number of amino acids, physical and chemical properties, subcellular localization, and secondary structure characteristics	Responsive to high-temperature and drought stresses in *N.franchetii*	(Wu et al., 2025)
	Dicotyledoneae *Salvia miltiorrhiza*	Yes 61 LEA genes in seven groups	Various cis-acting elements associated with abiotic stress responses	Transcriptional induction by drought stress	(Chen et al., 2021)
	Dicotyledoneae *Gossypium hirsutumGossypium arboreumGossypium raimondii*	Yes 136–242 LEA genes in eight groups	Majority involved in secretory pathways	Responsive to drought tolerant	(Magwanga et al., 2018)
	Dicotyledoneae *Brassica napus*	Yes 108 LEA genes in eight groups	Transcriptional induction in leaves and late-stage seeds	Undetermined	(Liang et al., 2016)
	Dicotyledoneae *Vigna radiataVigna angularisVigna unguiculata*	Yes 55–82 LEA genes in eight groups	Upregulation in response to heat stress	Transcriptional induction in stress conditions	(Nagaraju et al., 2019)
	Dicotyledoneae *Carya illinoinensis*	Yes 43 LEA genes in seven groups	Certain LEAs enhancing tolerance to osmotic stress, desiccation and salinity	LEA4 as an early stress responder to stresses	(Pulicherla et al., 2025)
	Dicotyledoneae *Juglans mandshuricaJuglans regia*	Yes 20–25 LEA genes in eight groups	Mainly expressed in leaves, green husks, male and female flowers	Prediction of *JrLEAs* involved in anthracnose resistance	(Ma et al., 2023)
	Dicotyledoneae *Amaranthus cruentus*	Yes 126 LEA genes in seven groups	Transcriptional induction under various stresses	Transcriptional response to drought	(Ma et al., 2023)
	Dicotyledoneae *Populus trichocarpa*	Yes 88 LEA genes	Genomic conserved and homologous to related LEA genes in other species	Differential expression of *PtrLEA* in roots, stems and leaves in response to salinity	(Cheng et al., 2021)
	Dicotyledoneae *Cucumis meloCitrillus lanatus*	Yes 61 LEA genes in three groups (*Cucumis melo*) 73 LEA genes in four groups (*Citrillus lanatus*)	Transcriptionally expressed in root and leaf tissues in drought condition	ClLEA-12-17–46 and CmLEA-42–43 as early responsory with gene induction	(Celik Altunoglu et al., 2017)
	Dicotyledoneae *Solanum tuberosum*	Yes 74 LEA genes in nine groups	StLEA genes involved in development processes and abiotic stresses	Differential expressions in response to hormone and stresses (drought, salinity, heavy metal, heat and cold)	(Chen et al., 2019)
	Dicotyledoneae *Myricaria laxiflora*	Yes 31 LEA genes in nine groups	LEA genes involved in flooding-drought adaption	Responsive to anaerobic induction, ABA and chilling/light stress	(Wu et al., 2025)
	Dicotyledoneae *Carica papaya*	Yes 28 LEA genes in eight groups	Diverse expression patterns of CpLEA genes in various tissue and different stages of developmental fruit	Significantly regulated by drought, cold and salt stresses	(Zou et al., 2022)

To date, functional studies on LEA proteins have predominantly focused on angiosperms, including monocotyledon and dicotyledon. Whereas LEAs data for hornworts in Bryophyta, peridophyta and cycadopsida/ginkgoopsida in Gymnospermae remain undetermined or unreleased, due to insufficient systematic study (**Table 1**). Nevertheless, studies extend to non-agiosperm species: for instance, 35 LEA-like proteins have been identified in a liverwort (Hepaticae), exhibiting typical ABA- and drought-responsive characteristics (Jahan et al., 2019), while 23 LEAs have been characterized in *Pinus tabuliformis* from peridophyta, representing transcriptional heat- and salinity- responses (Gao and Lan, 2016). This temporal-and-spatial expression pattern under drought is observed across the vast majority of LEA proteins (Ding et al., 2021).

The function of LEA groups is not only restricted solely to dehydration protection, but also exhibit stable expression in specialized cells, tissue and organs, including spores (Yotsui et al., 2016) and seeds (Smolikova et al., 2020). These observations that certain LEA mRNAs accumulate to high concentration and become uniformly distributed throughout the cytosol of all embryonic cell types warrant consideration (Roberts et al., 1993). Notably, the responsive expression pattern of LEA proteins within embryo during seed development exhibits marked similarity to that observed when plants are exposed to environmental dehydration (Bartels et al., 2025). During seed development and maturation, the absence of specific LEA proteins, DHNs can cause impaired seed viability (Aziz et al., 2021). Nevertheless, mechanistic studies are lacking for most of the listed LEA proteins/genes (**Table 1**), which limits the functional understanding of LEAs either against stress stimuli, or embryogenesis development. Although previous studies suggest that these post-translational LEA molecules may serve as hydration surrogate, stabilizing the semidry intracellular surface of cells within seeds (Hernandez-Sanchez et al., 2022). Their characteristic amino-acid composition allows LEA proteins self-stabilization (Battaglia et al., 2008) and chaperone activity across diverse environments (Amara et al., 2014).

## 4 LEA genes/proteins in bryophyte-grade ancestor *P. patens* bridging embryogenesis and abiotic stress responses

Bryophytes retain core ancestral traits of early land plants, making them an ideal evolutionary model systems for dissecting both vegetative and productive processes (Haig, 2016). Bryophytes comprise three major groups: mosses, liverworts, and hornworts. Among these, mosses represent unique developmental features, including the dominance of the vegetative growth, from single-celled protonemal filaments to leafy shoots of the mature gametophyte (Ligrone et al., 2012). Under stress conditions, such as nutrition limitation or short-day condition and low temperature, sporophytes can be promoted to generate the sexual organs: the antheridia and archegonia (Yoro et al., 2023). Following fertilization, embryo development is initiated upon zygote formation. Owing to its pivotally phylogenetic position anterior to vascular plants, combined with its distinctive anatomical and morphological traits, *P. patens* show outstanding advantages as a model organism for evolutionary development model (Yoro et al., 2023). Meantime, like other mosses, *P. patens*, exhibits a haplodiplontic life cycle, featuring a regular alternation between the diploid sporophyte and the haploid gametophyte (Rensing et al., 2020).

Following spore germination, *P. patens* produces tip-growing protonema comprising chloronema and caulonema (Jang and Dolan, 2011), which contain different amounts of chloroplasts (Pressel et al., 2008). Developmental signals trigger the transition from filamentous growth to 3-D tissues, which produce leafy gametophores. Subsequent sexual organ formation enables fertilization, which develops into an embryo and matures in sporophyte to finish the life cycle of *P. patens* (Cove and Knight, 1993). Fesenko et al. (2015) collected samples from different developmental stages of *P. patens*, along with protoplast samples, and performed transcriptional profile studies and quantitative analysis. Five out of six *PpLEA* genes are detected to represent a large amount of expression induction in gametophore, compared to those in protonema during the early stage (**Supplementary Table 1**). The expression of LEA proteins tends to reach higher levels in gametophores than in protonema or protoplasts, this pattern that likely reflects a protective role against dehydration. Unlike protonema, most gametophores are aerial tissues, where water homeostasis is critical. Additionally, gametophores are responsible for supporting fertilization and zygote formation. Another study compares transcriptional profiles in gametophyte and sporophyte in early or mid-stage. Unexpectedly, most *PpLEA* genes do not exhibit differential expression, with only one exception, which indicates that their expressions remain relatively high from gametophyte stage through embryo formation to early and mid-sporophytes (O'Donoghue et al., 2013). The single *PpLEA* gene showing differential expression during the gametophyte-to-sporophyte transition is activated at this developmental onset and maintains comparable expression thereafter (**Supplementary Table 2**), suggesting its unique and non-redundant function linking vegetative and reproductive developmental stages. Accumulated evidence confirms that PpLEAs participate in vegetative and productive processes through P. patents life cycle (**Figure 2**). Presumably, PpLEA genes/proteins accumulate to protect gametophore development from desiccation, and this protection likely extends to the reproductive stages, ensuing zygote formation even during drought.

**Figure 2:**
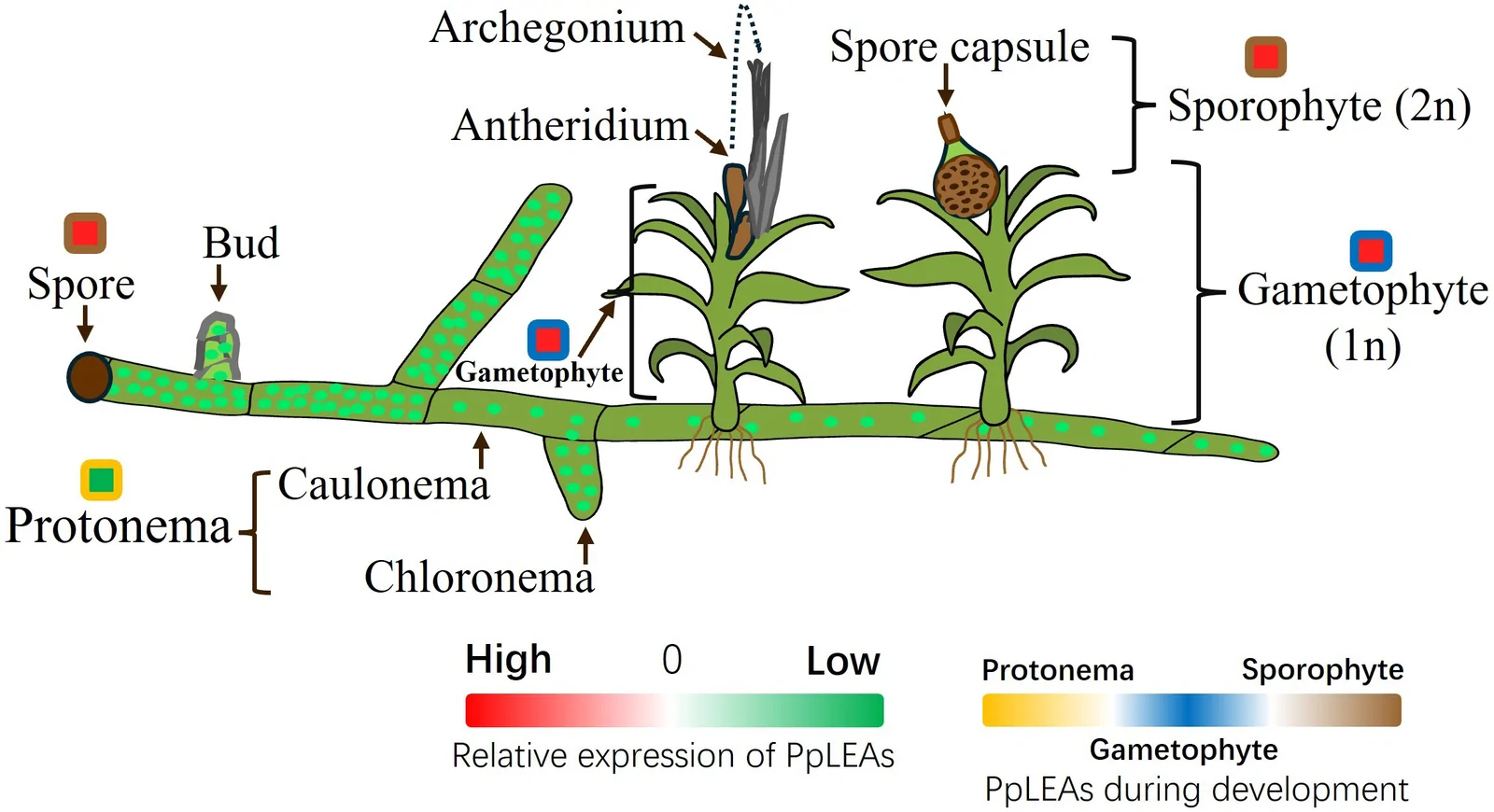
PpLEAs participate in vegetative and productive processes during *P. patens* life cycle.

*P. patens* biosynthesize LEA proteins in response to drought, chilling or abscisic acid (ABA) and etc (Minami et al., 2005; Wang et al., 2009; Cui et al., 2012; Yotsui et al., 2016). To avoid damage from various stressors, several LEA proteins either interact with biological membranes to protect their integrities and stabilities, or undergo structural transitions that confer appropriate protection under adverse conditions (Ruibal et al., 2013). Evidence from multiple experimental approaches supports the hypothesis that these LEA proteins exert broad aggregation-based protection, rather than specifically safeguarding any particular protein. However, information regarding the interaction and binding mechanisms between LEA proteins and cellular membranes remains limited. Nonetheless, several studies propose that the accumulation of certain LEA proteins promotes increased lipid desaturation in the cytomembrane. This effect is achieved either through reduced lipid diffusion coupled with repressed peroxidation, or via biophysical stabilization of the bilayer membrane—modulating membrane curvature internally and/or suppressing membrane crosslinking externally (Hernandez-Sanchez et al., 2022).

Owing to the full genome sequence of *P. patens* was released several years ago (Rensing et al., 2008), several milestone studies have been published since then. Although Alexander F. Routh group had performed bioinformatic analysis and identified 47 *PpLEAs* in *P. patens*, which was attached in the Supplementary (Yuen et al., 2019). However, in 2024, the gap-free genome was released, which was generated and assembled using 3rd generation sequencing methodology. Moreover, this telomere-to-telomere genome (Version 6.1) updates its chromosome number at 26 (Bi et al., 2024). Thus, the whole genome-wide identification, expression profiling, and detailed targeted identification of *PpLEA* genes are even more essential for the verification and/or further data mining, which hinders subsequent functional studies. Due to limited research released, the puzzle of PpLEA proteins in bridging embryogenesis and vegetative development under abiotic stress remains an open question. Nevertheless, *P. patens*—as a comparatively well-studied moss and an ideal model organism—may offer a valuable system for elucidating the connection between these two developmental programs, even under diverse stress conditions.

## 5 Discussion

The challenges of environmental and non-environmental stresses represent constantly severe impacts on biomass. Therefore, it is crucial to understand underlying plant response-adaption pattern and mechanisms. The *LEA* genes family, which plays a crucial role in plant stress responses, has been well characterized in Angiospermae, such as *Arabidopsis*, *Sorghum*, *Zea* and *Vitis*. Despite the impressive progress on *LEA* genes/proteins have been achieved in recent years, far less is known about their counterparts in Gymnospermae, Pteridophyta or Bryophyta. These missing pieces represent important knowledge gaps that offer valuable opportunities to unravel the intricate and nuanced mechanisms operating in resurrection plants.

This mini-review summarizes previous and current studies on the *LEAs* in embryophytes, mainly focusing on summary together with our understanding of certain *PpLEA* genes during vegetative and productive processes in *P. patens* (Bryophyta). Although 47 *PpLEAs* have been reported several years ago based on the data of next-generation sequencing, which emphasized the evolutionary conservation and functional significance of the *LEA* family. Nevertheless, comprehensive genome-wide identification of *PpLEA* genes would be essential based on the recently released telomere-to-telomere genome, which potentially provide more resources for future studies. Notably, *PpLEAs* are not only detected consistently in vegetative tissues/organs (gametophores), but also in reproductive organs, even lasting to reproductive phase forming zygotes in sporophytes. Moreover, the extensive response of *PpLEAs* toward abiotic stresses throughout the whole cell cycle, together with expression accumulated in resurrection *P. patens* during reproductive phase, might relate to similar mechanisms as in “seed plants”. The envision of PpLEA proteins are the comprehensive linkage between stress conditions and embryo development, mimicking the transition from vegetation to production process. To be summarized, the roles of *PpLEAs* are unrestricted in stress responses, which can be further exploited in multifaceted ways.
