# Supplementary material for: PpLEA genes in embryophytes,instead of LEAs being angiosperm-unique
Source: Front Plant Sci. 2026 Sep 15;17:1917865. doi: 10.3389/fpls.2026.1917865 (PMC13620466; doi:10.3389/fpls.2026.1917865)
Supplement: Supplementary file 1 [file DataSheet1.pdf]

PpLEA genes in Embryophytes, instead of LEAs being Angiosperm-unique

Jing Du[1*], Ji Li[1], Lei Wang[1*]

[1]Key Laboratory of Xinjiang Phytomedicine Resource and Utilization of Ministry of Education, Xinjiang Production and Construction Corps Key Laboratory of Oasis Town and Mountain-basin System Ecology, College of Life Sciences, Shihezi University, Shihezi, Xinjiang, 832003, China

Supplementary

Supplementary Table 1. Summary List of PpLEA proteins and peptides identified in gametophores, protonema and protoplast.

**Accession**		A9S6V7	A9SG80	A9SSU4	A9SU24	A9SU25	A9TMN8
**Gene name**		/	/	/	LEA3, LEA	/	LEA3, LEA
**Total number of identified peptides mainly in Gametophore**		15	16	22	45	30	42
**Expression (RPKM)**	Gametophore	232.50	457.00	323.74	/	1140.25	1079.83
	Protonema	3.44	18.68	0.83	/	10.41	32.15
	Protoplast	8.16	80.28	4.72	/	2.47	85.06
**Relative expression ratio** (Gametophore/ Protonema)		**67.59**	**24.46**	**390.81**	**/**	**109.53**	**33.59**

RPKM : Reads Per Kilobase of exon model per Million mapped reads.

Supplementary Table 2. The single PpLEA gene with differential expression during transition from gametophyte to early and mid-sporophyte.

**Gene information**	**Gametophyte**	**Early sporophyte**	**Mid-sporophyte**

<table>
<tr><td rowspan="1">Sequence name</td><td>6064|gw1.234.26.1

XP_001778223(NCBI accession number)</td><td rowspan="3">LEA gene</td><td colspan="2">Differential expression

Supplementary Table S15</td><td></td></tr>
<tr><td rowspan="2">Description</td><td rowspan="2">late embryogenesis abundant domain-containing protein</td><td></td><td colspan="2">Non differential expression

Supplementary Table S17</td></tr>
<tr><td>Differential expression (vs mid-sporophyte)

Supplementary Table S16</td><td></td><td>Differential expression

(vs gametophyte)

Supplementary Table S16</td></tr>
<tr><td>Reference</td><td colspan="5">(O'Donoghue, Chater et al. 2013)</td></tr>
</table>

O'Donoghue, M. T., C. Chater, S. Wallace, J. E. Gray, D. J. Beerling and A. J. Fleming (2013). "Genome-wide transcriptomic analysis of the sporophyte of the moss Physcomitrella patens." J Exp Bot **64**(12): 3567-3581.
